# Z-score differences based on cross-sectional growth charts do not reflect the growth rate of very low birth weight infants

**DOI:** 10.1371/journal.pone.0216048

**Published:** 2019-05-07

**Authors:** Niels Rochow, Erin Landau-Crangle, Hon Yiu So, Anna Pelc, Gerhard Fusch, Jan Däbritz, Wolfgang Göpel, Christoph Fusch

**Affiliations:** 1 Department of Pediatrics, McMaster University, Hamilton, Ontario, Canada; 2 School of Medicine, Queen’s University, Kingston, Ontario, Canada; 3 Department of Statistics and Actuarial Science, University of Waterloo, Waterloo, Ontario, Canada; 4 Department of Pediatrics, University Hospital Rostock, Rostock, Germany; 5 Department of Pediatrics, University of Lübeck, Lübeck, Germany; 6 Department of Pediatrics, Paracelsus Medical University Nuremberg, Nuremberg, Germany; Center of Pediatrics, GERMANY

## Abstract

**Objective:**

To test whether the assessment of growth in very low birth weight infants during the hospital stay using z-score differences (Z_diff_) is confounded by gestational age (GA), birth weight percentiles (BW%ile), and length of the observation period (LOP). We hypothesize that Z_diff_ calculated from growth charts based on birth weight data introduces a systematic statistical error leading to falsely classified growth as restricted in infants growing similarly to the 50^th^ percentile.

**Methods:**

This observational study included 6,926 VLBW infants from the German Neonatal Network (2009 to 2015). Inclusion criterion was discharge between 37 and 41 weeks postmenstrual age. For each infant, Z_diff_, weight gain velocity, and reference growth rate (50^th^ percentile Fenton) from birth to discharge were calculated. To account for gestational age dependent growth rates, assessment of growth was standardized calculating the weight gain ratio (WGR) = weight gain velocity/reference growth rate. The primary outcome is the variation of the Z_diff_-to-WGR relationship.

**Results:**

Z_diff_ and WGR showed a weak agreement with a Z_diff_ of -0.74 (-1.03, -0.37) at the reference growth rate of the 50^th^ percentile (WGR = 1). A significant proportion (n = 1,585; 23%) of infants with negative Z_diff_ had weight gain velocity above the 50^th^ percentile’s growth rate. Z_diff_ to WGR relation was significantly affected by the interaction of GA x BW%ile x LOP.

**Conclusion:**

This study supports the hypothesis that Z_diff_, which are calculated using birth weights, are confounded by skewed reference data and can lead to misinterpretation of growth rates. New concepts like individualized growth trajectories may have the potential to overcome this limitation.

## Introduction

**Fig 1:**
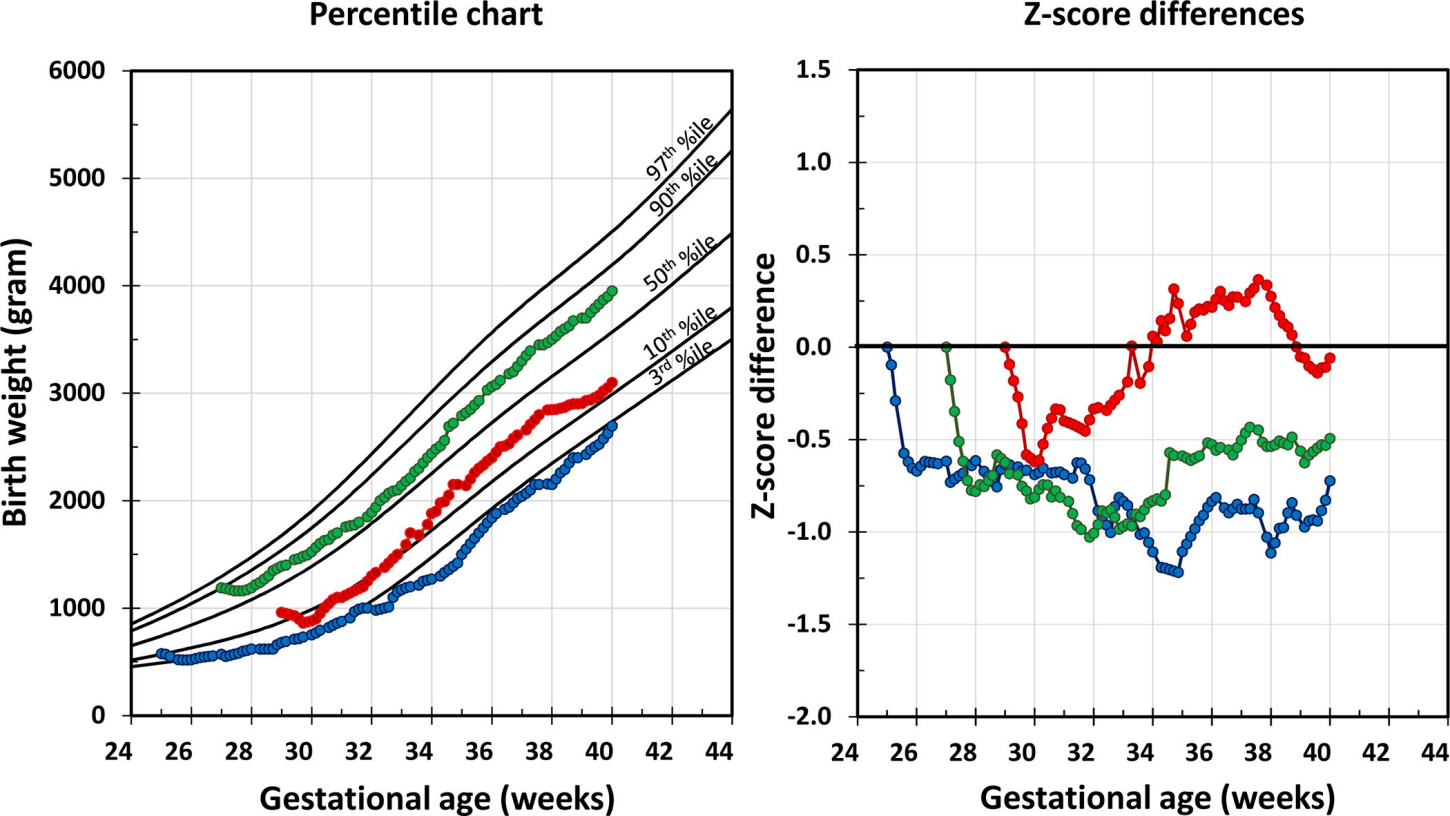
Common tools for the assessment of growth: percentile charts (left panel) and z-score plots (right panel), modified from Fenton et al. 2013 and Griffin et al. 2014; colored lines and dots represent growth trajectories of three example infants.

Changes in percentiles or z-scores during defined observation periods, such as from birth to discharge, are commonly used to assess growth of preterm infants. Tools presently applied in clinical routine for monitoring and guiding growth include growth charts and z-score plots (Fig 1).[1–3]

**Fig 2:**
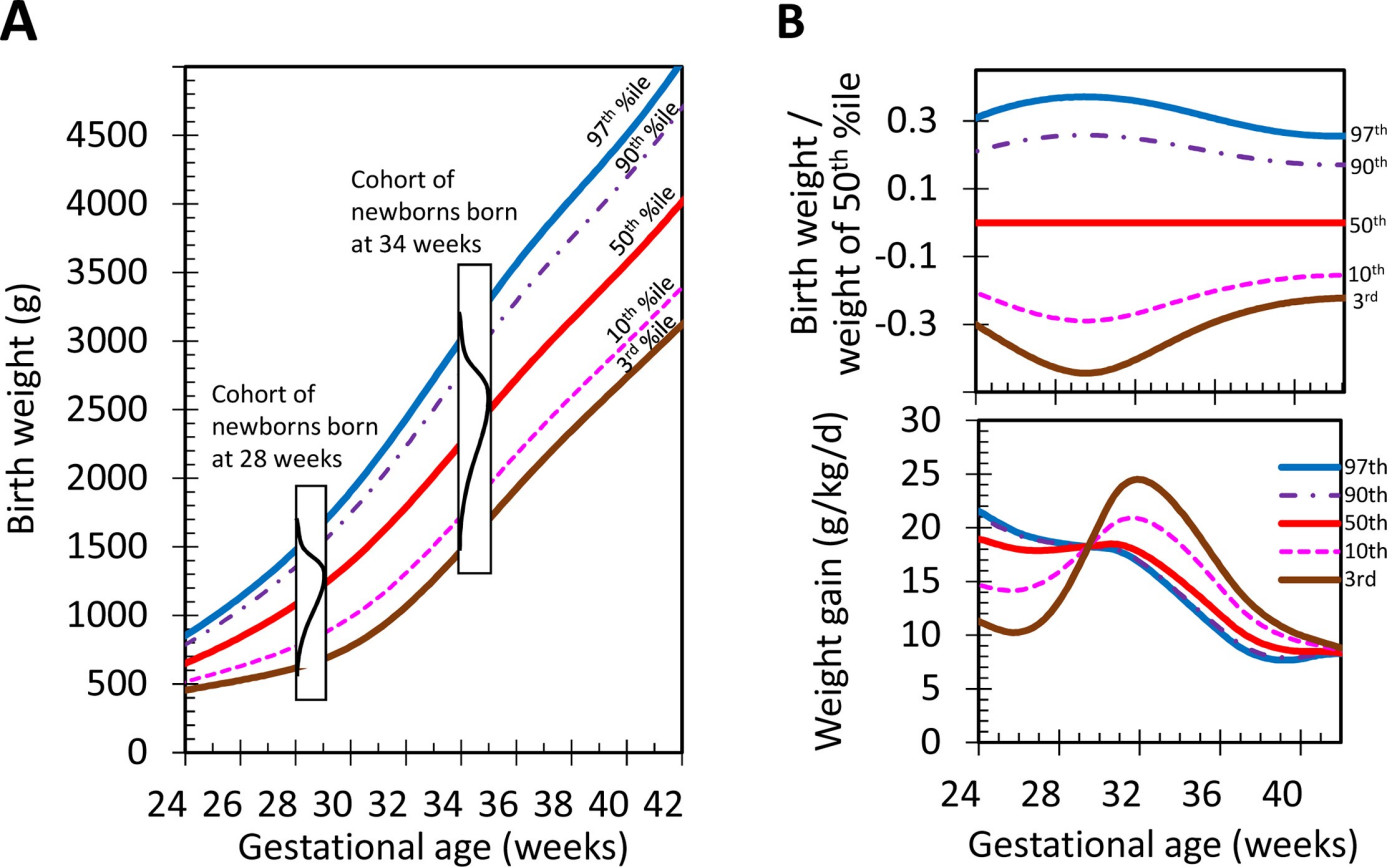
2A: Plotting of growth charts and calculation of z-scores, distribution (black line) of birth weights for a cohort of newborns with a gestational age of 28 and 34 weeks; 2B: Characteristics of growth charts: skewness of percentile distributions (top) and weight gain velocity for major percentiles (bottom).

Cross-sectional data from pregnancies with known birth weights and gestational ages serve as the basis for percentile growth charts. A percentile is defined as the birth weight value below which a given percentage of observations in a selected cohort can be found. For example, the 10^th^ percentile for a gestational age of 28 weeks is the birth weight value below which 10% of the neonates in the cohort fall at 28 weeks. Percentiles of birth weights for each gestational week are calculated using datasets of infants born at given weeks (e.g. in the 27^th^, 28^th^ or 34^th^ week) (Fig 2).[4]

Once birth weight percentiles have been built from the different cohorts, a growth chart is subsequently created by connecting these percentile values across gestational ages (e.g. from 24 to 42 weeks). However, linking distributions of percentile values between the weeks of gestation results in trajectories that are not representative of physiological growth.[5] Nonetheless, these trajectories are used in clinical practice as physiological growth curves in order to monitor growth and adjust feeding.

Z-scores are another commonly used parameter to compare individual weights with the cohort.[2, 6] A z-score (or a standard deviation score) is the number of standard deviations an individual weight is above or below the mean birth weight value. The z-score for a particular gestational age and sex is defined as:

$$Z–score=\frac{\left(individual\;weight-cohort\;mean\right)}{cohort\;standard\;deviation}$$

For instance, a z-score of -1 depicts an individual birth weight that is one standard deviation below the mean birth weight of the cohort. Z-score differences (Z_diff_) are used to analyze changes in growth for a particular observation period. Z_diff_ is defined as:

$$Z–diff=\left(z–score_{t2}\right)-\left(z–score_{t1}\right)$$

(t1=startoftheobservationperiod,t2=endoftheobservationperiod)

Z-scores and standard deviations have been defined for normally distributed data. If the distribution of birth weight data at different gestational ages is skewed, the accuracy of the z-score during particular observation periods, and thus its utility in monitoring growth, are impacted. This becomes even more significant if the skewness changes during the observation period.

Unfortunately, varying degrees of skewness across gestational ages is significantly present in most birth weight charts used for growth analyses nowadays. For the preterm period, the datasets inherently include a substantially high number of pregnancies and preterm births that are affected by pathologies, thus leading to the preterm termination of the pregnancy. This severely impacts the accuracy of the fetal growth data, resulting in a skewed distribution of birth weight percentiles mostly towards lower percentiles. As can be seen in Fig 2, Panel B (top), the relative distance between percentiles and standard deviations (standard deviation/mean) varies over the range of gestational ages. For example, the relative distance between the 3^rd^ percentile (brown line) and the 50^th^ percentile (red line) ((50^th^ percentile - 3^rd^ percentile) / 50^th^ percentile) increases up to 29 weeks, followed by a decrease until term age.[1, 3, 6] Despite this unexplained variation, target growth trajectories of preterm infants are estimated by using these percentile lines in routine neonatal care.

Fig 2, Panel B (bottom) depicts a set of resulting weight gain velocities (g/kg/d) when growth trajectories follow major percentiles (3^rd^, 10^th^, 50^th^, 90^th^ and 97^th^) on standard growth charts.[1] Considerable fluctuation in weight gain velocity can be seen, especially when following the 3^rd^ and the 10^th^ percentile curves. From a physiological perspective however, it is not evident why an infant should undergo such a weight gain velocity fluctuation during the last trimester of gestation. Moreover, the cause for the inverse order of the fluctuation seen for the 3^rd^ and 10^th^ percentile compared to the curves of the 50^th^ or the 90^th^ percentiles is unclear. These findings illustrate that the variation of skewness of percentiles across gestational ages might impact the accuracy of the Z_diff_ calculation.

We hypothesize that applying the current Z_diff_ approach will introduce a systematic error and may indicate growth restriction in preterm infants that are in fact growing at median rates of reference charts. Therefore, the objective of the current study is to investigate whether the assessment of growth of preterm infants by Z_diff_ is affected by the parameters gestational age, birth weight percentile, or by the length of the observation period. Mathematical models will be used to test the impact of the aforementioned three parameters on the Z_diff_.

## Methods

This observational study was performed using a data set of very low birth weight (VLBW, birth weight < 1,500 g) infants from the German Neonatal Network (2009 to 2015). All VLBW infants that were discharged between 37 to 41 weeks were included in this study. The study was approved by the Hamilton Integrated Research Ethics Board (HiREB) (2016-1696-C). The data were anonymized, and ethics committee waived the requirement for informed consent for this retrospective study.

**Fig 3:**
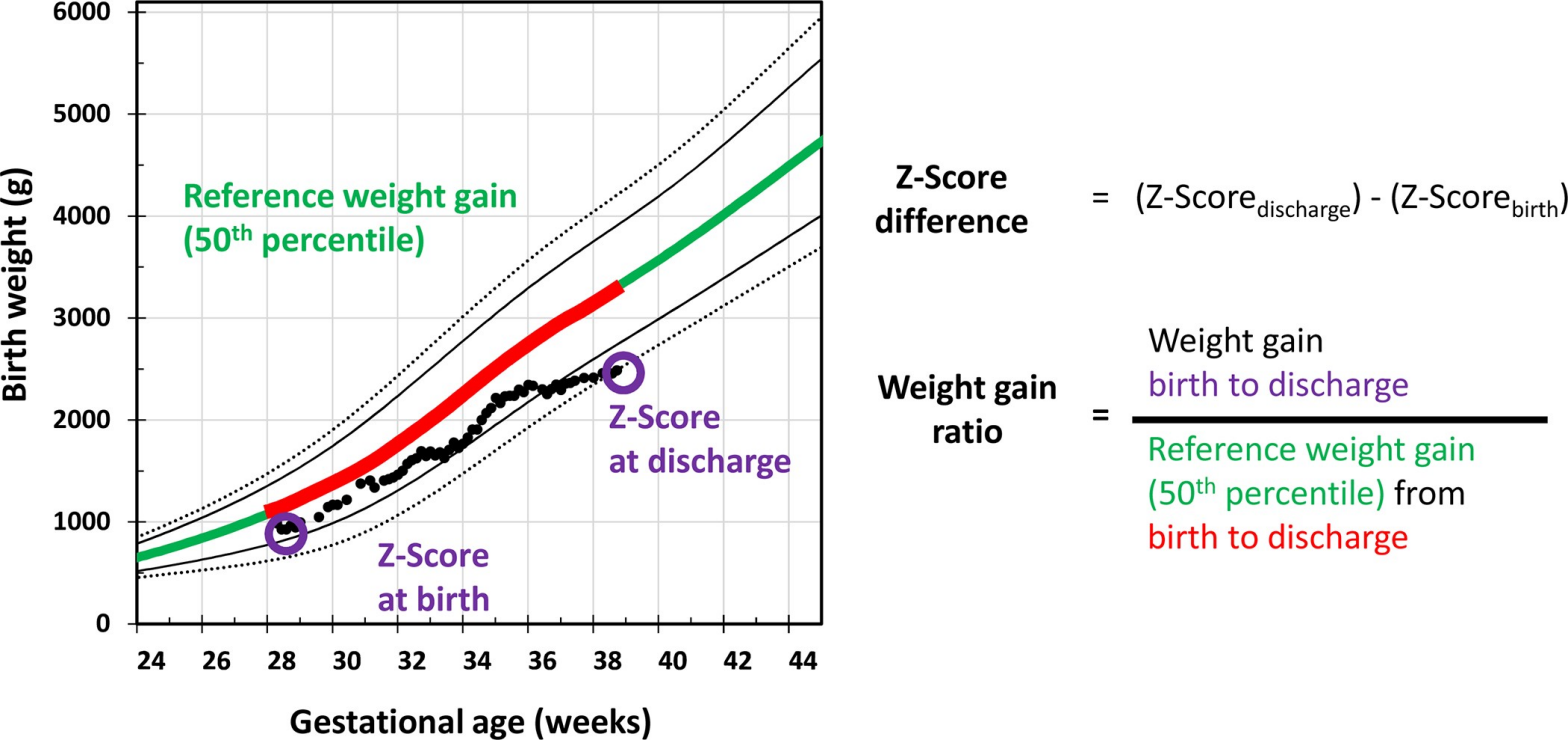
Method for the analysis of z-score differences in relation to weight gain velocity; the left panel shows how the reference data for weight gain velocity and z-score calculation were selected; the right panel shows the corresponding equations for z-score difference and weight gain ratio calculation.

For each infant, the z-scores for weight at birth and weight at discharge were obtained using the LMS parameter from the Fenton chart to calculate the Z_diff_ (Fig 3).[1]

The z-scores were calculated using the equation:[7]

$$zscore=\frac{{\left(weight/M\right)}^{L}-1}{L∙S}$$

In order to account for gestational age dependent growth rates, assessment of growth was standardized by calculating the individual weight gain ratio (WGR). Adopted from recent approaches to growth analysis, the median weight data of intrauterine growth charts were used to standardize the WGR.[8] The WGR was defined as a ratio of the individual weight gain velocity from birth (t1) to discharge (t2) divided by weight gain velocity from reference data (50^th^ percentile) for the same gestational age period (Fig 3).

The individual weight gain velocity as well as the reference weight gain velocity were calculated using the following equation:[9]

$$weight\;gain\;velocity\;[g∙{kg}^{-1}∙d^{-1}]=ln\;\left(\frac{{weight}_{t2}}{{weight}_{t1}}\right)÷(t2-t1\;[d])∙1,000[g∙{kg}^{-1}]$$

(t1=startoftheobservationperiod,t2=endoftheobservationperiod)

WGR is described using the following equation:

$$WGR=\frac{{weight\;gain\;velocity}_{individual}}{{weight\;gain\;velocity}_{reference}}$$

The reference data for the 50^th^ percentile used were obtained from the Fenton growth chart 2013.[1]

The primary outcome is the degree of variation of the Z_diff_-to-WGR relationship (homogeneity).

The effects of gestational age, birth weight percentile, and length of the observation period on the Z_diff_ were analyzed using regression models employing the r statistic function lm.

Model 1 tested the overall relationship between Z_diff_ and WGR using a linear regression analysis.

$$lm(Z_{diff}∼WGR)$$

Model 2 analyzed the effects of the three parameters (gestational age (GA), birth weight percentile (BW%ile), and length of the observation period (LOP)) on the correlation with Z_diff_.

$$lm(Z_{diff}∼WGR+GA+BW\%ile+LOP)$$

The third model tested whether WGR, GA, BW%ile, and LOP affects the relationship between the Z_diff_ and the WGR. This model tested whether the interaction of the parameters had an amplified effect, compared to when the parameters were considered independently (Model 2). It was hypothesized that Z_diff_ are non-linearly related to WGR, GA, BW%ile, and LOP. Model 3 was defined as follows:

$$lm(Z_{diff}∼WGR*GA*BW\%ile*LOP)$$

The difference between the models was analyzed using ANCOVA. The level of significance is p<0.05. The analysis was assisted by R software package for statistical analysis, R Foundation for Statistical Computing, version 3.5.0 (2018-04-23), Vienna, Austria.

## Results

This study was comprised of 6,926 (male n = 3,461; 50%) VLBW infants. The patient characteristics are outlined in Table 1.

**Table 1:** Patient characteristics.

		All	Gestational age group		
			≤27 weeks	28–31 weeks	≥32 weeks
Birth (t_1_)	N	6926	2539	3293	1094
	GA (weeks)	29.0 ± 2.8	26.1 ± 1.2	29.8 ± 1.1	33.3 ± 1.1
	Weight (g)	1070 ± 280	820 ± 200	1170 ± 220	1340 ± 150
	SGA n (%)	1594 (23.0)	297 (11.7)	497 (15.1)	800 (73.1)
	LGA n (%)	168 (2.4)	140 (5.5)	28 (0.9)	0 (0)
	z-score birth (t_1_)	-0.5 ± 1.0	-0.1 ± 0.9	-0.5 ± 0.7	-1.7 ± 0.6
	Length (cm)	36.6 ± 3.6	33.6 ± 2.8	37.9 ± 2.8	39.8 ± 2.4
	HC (cm)	25.9 ± 2.5	23.6 ± 1.9	26.8 ± 1.7	28.5 ± 1.3
Discharge (t_2_)	PMA (weeks)	38.7 ± 1.3	39.2 ± 1.4	38.5 ± 1.2	38.4 ± 1.1
	Weight (g)	2620 ± 450	2780 ± 480	2630 ± 410	2250 ± 290
	z-score difference (t_2_-t_1_)	-0.9 ± 0.8	-1.2 ± 1.0	-0.8 ± 0.7	-0.5 ± 0.5
	Weight gain velocity (t_2_-t_1_) (g/kg/d)	13.8 ± 2.4	13.6 ± 2.0	13.7 ± 2.4	14.7 ± 2.8
	Weight gain ratio	1.0 ± 0.2	0.9 ± 0.1	0.9 ± 0.2	1.2 ± 0.3
	Length (cm)	46.2 ± 2.7	46.6 ± 2.9	46.4 ± 2.6	44.7 ± 2.3
	HC (cm)	33.0 ± 1.7	33.2 ± 1.8	33.2 ± 1.6	32.2 ± 1.4
Outcome	Sepsis	1884 (27.2)	678 (26.7)	883 (26.8)	323 (29.5)
	IVH grade 3–4	371 (5.4)	148 (5.8)	161 (4.9)	62 (5.7)
	ROP grade 3–5	330 (4.8)	111 (4.4)	172 (5.2)	47 (4.3)
	NEC stage 2–3	143 (2.1)	91 (3.6)	42 (1.3)	10 (0.9)
	severe BPD	278 (4)	104 (4.1)	140 (4.3)	34 (3.1)

GA-gestational age, PMA-postmenstrual age, HC-head circumference, t_1_ –birth, t_2_ –discharge

When using the linear regression model (Model 1), the following relationship between Z_diff_ and WGR was obtained:

$$Z_{diff}=-3.90+3.16∙WGR\;(R^{2}=0.55,\;p<0.0001)$$

**Fig 4:**
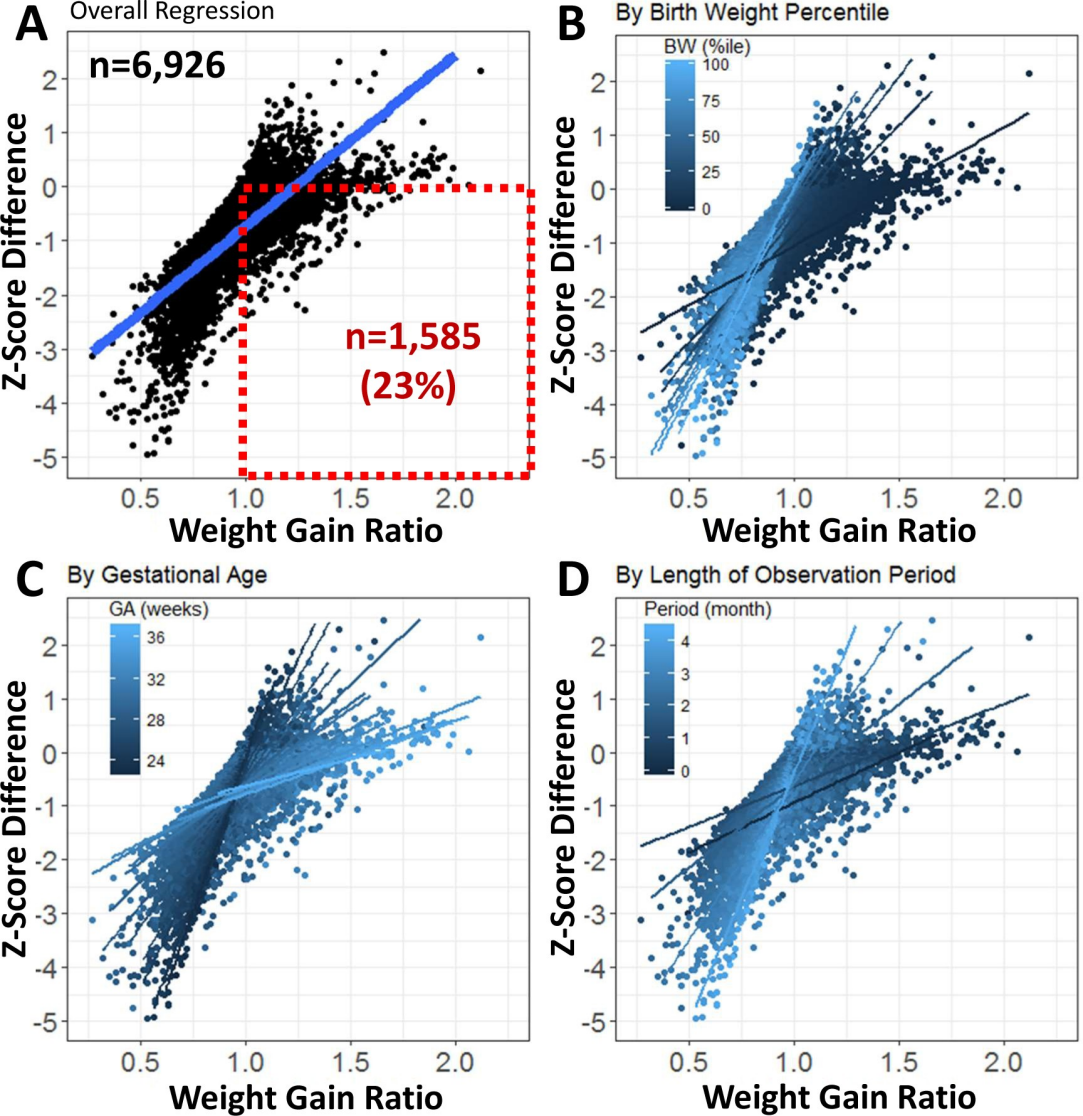
Longitudinal assessment of growth: Z_diff_ versus WGR relationship. Scatter plots show individual data and the regression line calculated for the complete data set (panel A), as well as a set of regression lines stratified by birth weight (panel B), gestational age (panel C) and length of observation period (panel D), colours represents stratification for four different parameters as indicated in legends. Dotted red box (panel A) shows proportion of infants which show a WGR>1 and negative z-score.

This analysis revealed that at a WGR = 1, which should correspond to a growth rate following the 50^th^ percentile, the Z_diff_ was -0.74 at the regression line. The first and third quartiles of Z_diff_ were -1.03 and -0.37, respectively. As can be seen in Fig 4, Panel A, there is a significant proportion of infants n = 1,585 (23%) which show a WGR > 1, indicating growth faster than the 50^th^ percentile, when Z_diff_ is calculated, the result is negative. In contrast, the number of infants with WGR ≤ 1 and positive Z_diff_ is 18 (0.3%).

This subset of infants is highlighted by the dotted red box in Fig 4, Panel A. These infants are growing at the median rate, but would be classified as growth restricted because of this negative Z_diff_. Moreover, there was also a high inter-individual variation in the Z_diff_ of infants with the same WGR. The Z_diff_ differed by up to 4 z-scores for the same WGR (Fig 4).

The shape of the residuals plot of the linear model (Model 1) demonstrated a non-random distribution with a residuals standard error of 0.56 Z_diff_ (S1 Fig) indicating that additional factors affect the Z_diff_ and that the Z_diff_ is not linearly related to the WGR.

Fig 4 shows the effect of gestational age (GA), birth weight percentile (BW%ile), and length of the observation period (LOP) on the Z_diff_-to-WGR relationship. The slope of the regression lines for the infants with lower GA, BW%ile, and shorter LOP is steeper, which translates into larger changes of Z_diff_ with changes in WGR.

Model 2 revealed that gestational age, birth weight percentile, and length of the observation period have significant effects on the Z_diff_ (R^2^ = 0.60; p<0.0001). The comparison of Model 1 and Model 2 by ANCOVA showed a significant improvement of Z_diff_ prediction by Model 2 (F value = 236; p<0.0001).

$$Z_{diff}=3.56∙WGR-0.018∙GA+0.004∙BW\%ile-0.017∙LOP+0.28$$

Model 3 demonstrated that the interaction between the parameters (WGR, gestational age, birth weight percentile and length of the observation period) significantly affected the Z_diff_ (R^2^ = 0.95; p<0.0001).

The ANCOVA analysis revealed an increase in the explanatory power of Model 3 over 2 with an F value of 4,308 and a small p-value (p<0.0001). This indicates that Model 3 predicted Z_diff_ closer to the actual values in comparison to Model 2. The variation of the residuals also decreased when Model 3 was applied compared to Model 2. Thus, the interaction of the variables WGR, GA, BW%ile and length of the observation period has a stronger effect on the Z_diff_, compared to when these parameters are considered independently.

In summary, these mathematical findings imply that the relation between Z_diff_ and WGR is dependent on the variables gestational age, birth weight percentile, and length of the observation period. The analysis showed no sex-specific effects.

## Discussion

In this study, we found that z-score differences (Z_diff_) and weight gain velocity from birth to discharge do not correlate well in a significant portion of in VLBW infants. This finding supports the hypothesis that the approach of using Z_diff_ to assess growth in preterm infants is confounded by skewed reference data and thus, does not provide an accurate reflection of growth. Gestational age, birth weight percentile, and the length of the observation period have significant effects on the Z_diff_. The influence of these factors is supported by statistically significant mathematical findings. Thus, an unadjusted Z_diff_ cannot be accurately translated into growth rates or growth trajectories.

One explanation for the study’s findings could be that there is a significant variation of the standard deviation of birth weight data from early preterm to term age (Fig 2), leading to the paradoxical phenomenon that infants with the same growth rate have different z-scores. Between 28 and 30 weeks, the relative standard deviation (standard deviation divided by the mean) is twice the relative standard deviation at term gestational age.[3, 10] This widening affects the Z_diff_ calculation. For instance, assuming a mean weight of 1,000 g and a standard deviation of 200 g (~20%) at 28 weeks, an infant with a weight of 0.8 of the mean weight (800 g) would have a z-score of -1 (800 g– 1,000 g / 200 g). If this infant were to grow with the growth rate of the 50^th^ percentile, the infant would reach 0.8 of the mean weight by term age (2,720 g). The standard deviation at term age would be about half (~10% of the mean weight; 340 g). The z-score of this infant at term age would then be -2 (2,720 g– 3,400 g / 340 g) and the Z_diff_ would thus be -1. Another infant born at the mean weight and also growing with the growth rate of the 50^th^ percentile would have a z-score of 0 (1,000 g– 1,000 g / 200 g) at 28 weeks and a z-score of 0 at term age (3,400 g—3,400 g / 340 g), resulting in a Z_diff_ of 0 (S1 Table). Thus, even though both these infants are growing at the same rate, the z-score and Z_diff_ are different, illustrating that reliance solely on Z_diff_ for growth prediction and feeding adjustments is problematic.

In order for an infant to maintain an unchanged z-score, the infant would need to grow according to the fluctuating pattern of percentiles or standard deviations across the gestational weeks as depicted in Fig 2, Panel B (bottom). The variation is significant.[11] When expressed in percentiles, at 24 weeks of gestation, the ratio between the 3^rd^ and the 50^th^ percentile is 0.66. At 29, 34 and 40 weeks however, it is 0.57, 0.64 and 0.72, respectively. Refer to Fig 2, Panel B (top). There is, however, no physiological explanation justifying an infant following a fluctuating growth trajectory such as the ones observed for the 3^rd^ or the 10^th^ percentiles. This observation supports the claim that the Z_diff_ calculation and resulting Z_diff_ are dependent on the gestational age, and also on when the observation period for the given infant is complete.

The physiological condition of postnatal adaptation is not reflected by the z-score approach. Postnatal adaptation includes weight loss during the first few days of life, mostly due to an irreversible, one-time loss of extracellular water volume.[12–14] As can be seen in Fig 2, Panel A, absolute distances (in grams) between the birth weight percentiles for gestational age are similar between major percentiles (see distances between the 90^th^ and 97^th^ percentile, or between the 3^rd^ and 10^th^ percentile).[13, 14] During the period of postnatal adaptation, relative weight loss expressed as a percentage of birth weight has been found to be similar across infants of the same gestational age. Therefore, infants born at the same gestational age but in higher birth weight percentiles will cross more percentiles during the period of postnatal adaptation as compared to infants born at lower birth weight percentiles. An issue arises because in the z-score calculation, a fixed value for the standard deviation is applied to infants of different birth weights, experiencing a similar relative weight loss (e.g., 10%). For instance, an infant at the 97^th^ percentile with a birth weight of 1,430 g would undergo the 10% physiological weight loss, resulting in a weight of 1,290 g (74^th^ percentile). This infant has thus crossed 23 percentiles. Another infant at the 10^th^ percentile with a birth weight of 700 g undergoing the same 10% weight loss would result in a weight of 630 g (3^rd^ percentile). This infant has thus crossed only seven percentiles. The Z_diff_ would consequently be higher from birth to day 5 of life in infants with higher birth weight percentiles. Thus, this finding supports the hypothesis that birth weight percentile impacts Z_diff_.

In summary, the relation between Z_diff_ and weight gain velocity is weak when Z_diff_ is calculated within the postmenstrual age range from preterm (e.g. 24 weeks) to term age. This was shown in the current study using a large cohort of very low birth weight infants which stayed an average of 5 to 13 weeks in the NICU. We have identified that this finding is determined by the characteristics of the reference data used for z-score calculations. The reference data are cross-sectional birth weight data, which were developed to assess the nutritional status at birth. Employing these birth weight data for the analysis of postnatal growth diverges from the intended use of the data and introduces systematic errors in growth analysis.

The findings suggest that a novel approach to assess growth and rates of growth is necessary. A promising approach would be to apply the concept of individualized growth trajectories as recently proposed.[12, 15, 16] An individualized growth trajectory combines physiological considerations and data established for the different periods of growth after preterm birth. These periods include: 1) intrauterine growth until birth, 2) the physiological postnatal weight loss mainly due to contraction of extracellular water spaces and adjustment to the new postnatal trajectory, 3) stable growth and 4) transition to healthy term infant equivalent trajectories at post-term age. Individual growth trajectories would allow clinicians to calculate the absolute deviation, in grams, from the predicted weight trajectory at any given point in time. This would indicate to the clinician how well an individual infant is growing and allow for prompt adjustment of nutrition to achieve optimal growth.[12, 15, 16]

In conclusion, the calculation of z-scores, percentiles and Z_diff_ is dependent on the reference data. When birth weight data are used to assess longitudinal growth, the interpretation should be done with caution. Individualized growth trajectories are a promising alternative. Nonetheless, their use in clinical practice requires validation.

## Supporting information

S1 Fig (DOCX)

S1 Table (DOCX)
