# Supplementary figures and images for: Z-score differences based on cross-sectional growth charts do not reflect the growth rate of very low birth weight infants
Source: PLoS One. 2019 May 7;14(5):e0216048. doi: 10.1371/journal.pone.0216048 (PMC6504035; doi:10.1371/journal.pone.0216048)

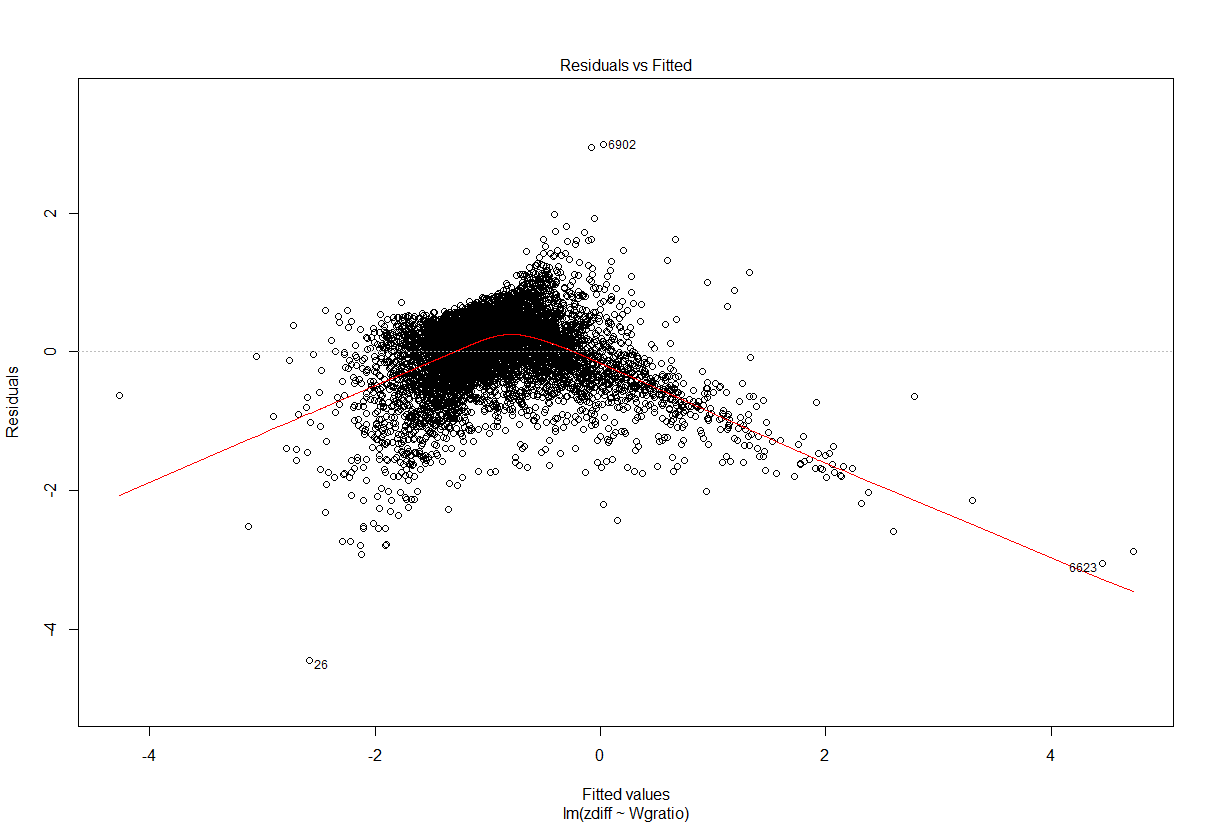


S1 Fig: Residuals versus fitted plot for the linear model Zdiff ~ Weight gain ratio

Supplement: S1 Fig — (DOCX) [file pone.0216048.s001.docx]
