# Supplementary material for: Z-score differences based on cross-sectional growth charts do not reflect the growth rate of very low birth weight infants
Source: PLoS One. 2019 May 7;14(5):e0216048. doi: 10.1371/journal.pone.0216048 (PMC6504035; doi:10.1371/journal.pone.0216048)
Supplement: S1 Table — (DOCX) [file pone.0216048.s002.docx]

S1 Table: Change of z-scores in preterm infants with the same weight gain.

| PMA (weeks) | Weight gain (g/kg/d) | Infant 1 (10^th^ %ile) | | | | | Infant 2 (50^th^ %ile) | | | | Infant 3 (90^th^ %ile) | | | | |
| --- | --- | --- | --- | --- | --- | --- | --- | --- | --- | --- | --- | --- | --- | --- | --- |
|  |  | Weight (g) | Weight ratio^$^ (Inf_1_/Inf_2_) | %ile | z-score | z-score diff. | Weight (g) | CV  (%) | %ile | z-score diff. | Weight (g) | Weight ratio^$^ (Inf_3_/Inf_2)_ | %ile | z-score | z-score diff. |
| 29 | 18 | 865 | 0.75 | 15 | -1.0 | 0 | 1152 | 23.0 | 50 | 0 | 1420 | 1.23 | 85 | 1.0 | 0 |
| 30 | 18.2 | 981 | 0.75 | 15 | -1.0 | 0 | 1306 | 23.1 | 50 | 0 | 1610 | 1.23 | 85 | 1.0 | 0 |
| 31 | 18.3 | 1113 | 0.75 | 14 | -1.1 | 0 | 1482 | 22.7 | 50 | 0 | 1827 | 1.23 | 85 | 1.0 | 0 |
| 32 | 17.8 | 1262 | 0.75 | 13 | -1.1 | -0.1 | 1681 | 21.9 | 50 | 0 | 2072 | 1.23 | 86 | 1.1 | 0 |
| 33 | 16.9 | 1425 | 0.75 | 11 | -1.2 | -0.2 | 1897 | 20.7 | 50 | 0 | 2339 | 1.23 | 87 | 1.1 | 0.1 |
| 34 | 15.7 | 1596 | 0.75 | 9 | -1.3 | -0.3 | 2126 | 19.4 | 50 | 0 | 2621 | 1.23 | 88 | 1.2 | 0.1 |
| 35 | 14.5 | 1774 | 0.75 | 7 | -1.5 | -0.4 | 2362 | 18.1 | 50 | 0 | 2912 | 1.23 | 89 | 1.2 | 0.2 |
| 36 | 13.1 | 1954 | 0.75 | 6 | -1.6 | -0.5 | 2602 | 17.0 | 50 | 0 | 3207 | 1.23 | 90 | 1.3 | 0.3 |
| 37 | 11.3 | 2129 | 0.75 | 5 | -1.7 | -0.6 | 2835 | 16.1 | 50 | 0 | 3495 | 1.23 | 91 | 1.3 | 0.3 |
| 38 | 9.4 | 2290 | 0.75 | 4 | -1.8 | -0.7 | 3050 | 15.5 | 50 | 0 | 3760 | 1.23 | 92 | 1.4 | 0.3 |
| 39 | 7.9 | 2433 | 0.75 | 3 | -1.9 | -0.8 | 3239 | 15.0 | 50 | 0 | 3993 | 1.23 | 92 | 1.4 | 0.4 |
| 40 | 7.4 | 2565 | 0.75 | 3 | -1.9 | -0.9 | 3415 | 14.7 | 50 | 0 | 4210 | 1.23 | 93 | 1.5 | 0.4 |

PMA – postmenstrual age, ^$^weight ratio shows the ratios for infant’s 1 weight divided by infant’s 2 weight and infant’s 3 weight divided by infant’s 2 weight,
CV is the coefficient of variation or S value of the LMS values in percent
